# Effects of Virtual Reality Therapy for Patients With Breast Cancer During Chemotherapy: Randomized Controlled Trial

**DOI:** 10.2196/53825

**Published:** 2024-10-17

**Authors:** Mengdan Li, Zhifu Yu, Hui Li, Li Cao, Huihui Yu, Ning Deng, Yunyong Liu

**Affiliations:** 1 Cancer Hospital of China Medical University, Liaoning Cancer Hospital & Institute, ShenYang, China; 2 National Cancer Center, National Clinical Research Center for Cancer, Cancer Hospital & Shenzhen Hospital, Chinese Academy of Medical Sciences and Peking Union Medical College, 113 Baohe Avenue, Longgang DistrictShenzhen, 518172, China, 86 18041381025

**Keywords:** virtual reality, breast neoplasms, quality of life, psychological distress, longitudinal studies

## Abstract

**Background:**

Patients with breast cancer endure high levels of psychological and physical pain. Virtual reality (VR) may be an acceptable, safe intervention to alleviate the negative emotions and pain of patients with cancer.

**Objective:**

We aimed to test the long-term effects of VR on psychological distress and quality of life (QOL) with traditional care in Chinese patients with breast cancer. We also explored the intervention mechanism and the acceptability of VR.

**Methods:**

A total of 327 eligible participants were randomly assigned to a VR intervention group or a control group. The Distress Thermometer, QLQ-C30 (Quality of Life Questionnaire version 3.0), and Virtual Reality Symptom Questionnaire were assessed at baseline, postintervention (3 mo), and follow-up (6 mo). Analysis followed the intention-to-treat (ITT) principle. The generalized estimating equations model was used to analyze the longitudinal data, and the PROCESS macro was used to analyze the mediating effect.

**Results:**

Compared with the control group, patients with breast cancer in the VR group had lower distress scores (*P*=.007), and higher health-related QOL scores (physical, role, emotional, cognitive, and social functioning) after 6 months (*P*<.05). Psychological distress had mediating effects on the longitudinal association between VR and the health-related QOL (indirect effect=4.572‐6.672, all *P*<.05).

**Conclusions:**

VR intervention technology may help reduce distress and improve QOL for patients with breast cancer over time. By incorporating a mediating analysis, we showed that the QOL benefits of VR intervention was manifested through positive effects on psychological distress risk factors.

## Introduction

Breast cancer is a common malignant tumor throughout the world [1]. With the progress of detection and treatment technology, the survival rate of patients with breast cancer has been greatly improved. However, increasing numbers of studies have also paid attention to the adverse psychological and physiological sequelae caused by breast cancer surgery or chemotherapy [2,3]. Patients with cancer are at several times the risk of psychological disorders due to lack of normal life, communication, and interaction [4-6]. Other symptoms related to chemotherapy such as nausea, vomiting, and anorexia are frequently reported [7]. These may affect treatment compliance and quality of life (QOL). From 2020, COVID-19 has been spreading around the world. In the process of fighting the pandemic, the normal operations of the hospitals were affected [8]. The postponement of re-examinations for some patients with cancer, interregional hospitalization, and self-isolation have all increased patients’ emotional distress [9]. In addition, patients have less access to the outside world and have lost normal social interactions. Psychological needs are even less satisfied, with considerably increased loneliness [10]. Without timely intervention and treatment, a poor mental state might cause tumor progression and deterioration [11].

Virtual reality (VR) is a 3D virtual scene that simulates reality, generated by a computer. It immerses the user in a virtual environment by using specific human-machine interfaces, such as a head-mounted display, a set of wired gloves, a position tracker or other controllers to experience a sense of presence or immersion [12]. Specifically, it is an artificial environment that is experienced through sensory stimuli such as images and sounds provided by a computer. In this environment, our behavior can partially determine our feelings [13]. In recent years, especially in the health system, the emergence of this technology provided patients with a safe environment for intervention and treatment [14-17]. Its effectiveness stemmed from the fact that patients could focus on pleasant or interesting stimuli rather than unpleasant symptoms [18]. These techniques were generally categorized as distraction interventions. By using head-mounted devices, VR immersed patients in computer-generated views while engaging various senses, providing a comprehensive stimulus that helped isolate patients from the hospital environment [19].

Previous studies have confirmed that the VR interventions could alleviate chemotherapy pain and enhance the QOL for patients with cancer [20]. However, research on the mediating mechanisms between VR and QOL remains limited. Among the potential mediating mechanisms, the most widely accepted hypothesis was that VR improves emotional states. Patients who experienced less anxiety, more fun, and more positive emotional valence during VR distraction were more likely to report subjective pain reduction [21]. Specifically, when patients experienced more enjoyment, painful treatments became more tolerable [22], potentially enhancing their self-efficacy and, consequently, improving their QOL [22,23]. These studies suggested that the relationship between VR and the QOL in patients with cancer might have been mediated by emotional regulation. Therefore, it is necessary to verify the effectiveness and obtain the best clinical practice evidence.

Some studies on VR had some limitations, for example, most of the trials lacked a control group [23,24], and the sample size was small (n<50) [25-27]. Owing to the lack of repeated exposure and long-term follow-up results [28-30], it has not been adequately established whether the effects of VR could endure for longer timespans beyond the usage of the device. Our main objective was to explore the long-term effects of VR in reducing distress and some chemotherapy symptoms, as well as improving the QOL for patients with breast cancer in China. Moreover, we further explored whether or not psychological distress mediated the effect of VR interventions on the health-related QOL.

## Methods

### Study Design and Setting

This study was a single-blinded randomized controlled trial, and was registered in the Chinese Clinical Trial Registry (registration ChiCTR2000035049). Patients were randomly assigned (1:1) to the VR group and control group by block randomization with varying block sizes of 4. The random sequence was generated using the Random Allocation Software (version 23; IBM Corp) by a graduate student who was not involved in the intervention or data collection. Sequentially numbered, opaque, sealed envelopes were prepared by the student. Before the first chemotherapy (baseline, T0), all the participants completed the informed consent form and a baseline questionnaire. Afterward, the second and third questionnaires were completed in the third month (post intervention, T1) and the sixth month (follow-up, T2), respectively. The content of the measurement includes both physical symptoms and psychological and emotional aspects.

### Participants

This study was conducted in the Cancer Hospital of China Medical University, which is a public cancer treatment center in northeastern China, from April 2020 to March 2021. The selection criteria were as follows: (1) a confirmed diagnosis and treatment of breast cancer for the first time, and a plan to undergo chemotherapy; (2) aged 18 to 79 years; and (3) complete cognition, normal reading ability, and barrier-free communication. The exclusion criteria were as follows: (1) doctors assessed life expectancy to be less than 6 months, (2) patients with emotional illness, and (3) patients who were receiving other forms of psychotherapy. All participants provided informed consent.

### Sample Size

The sample size was calculated using the G*Power (version 3.1; Heinrich-Heine-Universität Düsseldorf) program. Based on a 2-sided significance level of .05, an effect size of 0.6 for distress outcome [31], the 2-arm parallel trial power of 0.80, and a 35% attrition rate, a minimum of 130 participants per group was needed.

### Intervention Content and Frequency

**Figure 1.**
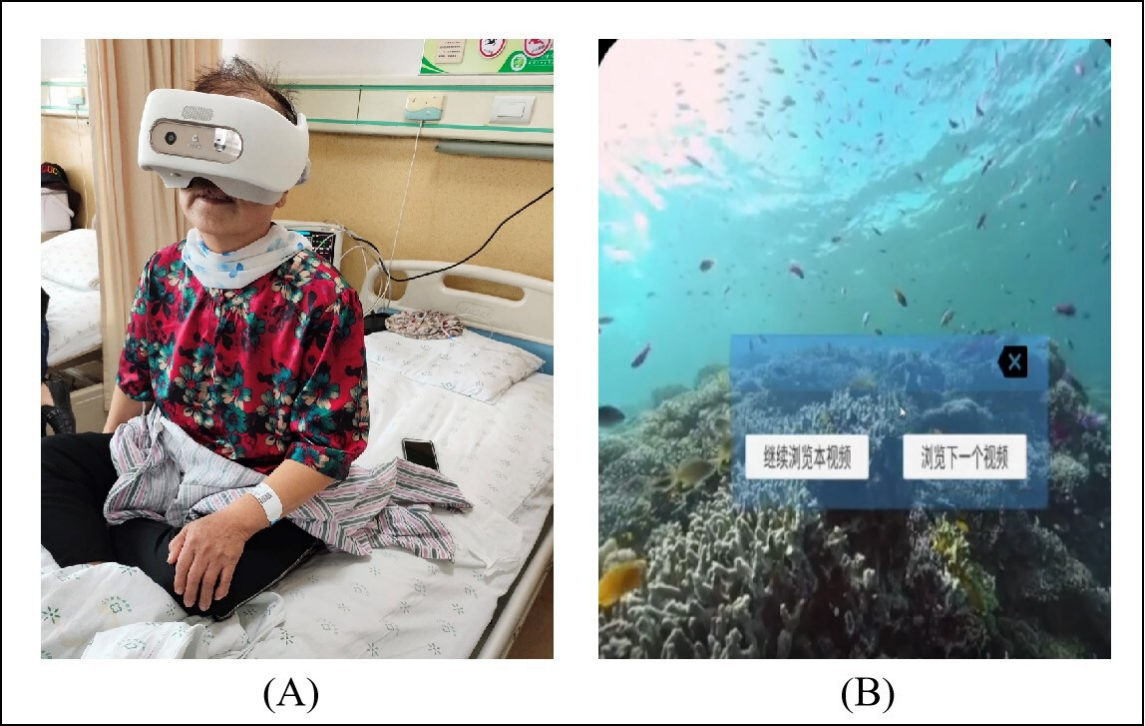
(**A**) shows how a virtual reality (VR) device was used during the intervention; (**B**) shows one of the screen captures from the VR experiences.

The VR device consists of 2 parts: a headset (VIVES110) and a hand controller. Our team developed 3 virtual environments in total for participants to choose freely, and embedded a voice guidance system in the device to help participants relax. Patients could complete explorations such as walking through the forest, walking on the beach, and sightseeing at sea, or relaxation training according to the voice guidance (Figure 1). The intervention trial was a 12-week VR training. The nurses guided participants to wear the device during chemotherapy intervals or during breaks, and to participate for 15‐20 minutes 1 to 2 times a week in the hospital. All participants had to have at least 12 interventions before T1. After each VR intervention, participants reported whether they had possible adverse symptoms. The participants in the control group only underwent chemotherapy as per the treatment plan in the hospital, and refrained from beginning any VR treatment.

### Measurements

#### Demographic and Clinical Information

Participants completed a standardized self-report in the first round of questionnaires, including age, residence, marital status, education level, employment status, and disease stage.

#### Distress

The Distress Thermometer (DT) [32] is a single-item, self-report measure of psychological distress. The DT has an 11-point range from 0 (no distress) to 10 (extreme distress). Patients were asked to choose the number that best describes how distressed they have been in the past week.

#### Health-Related QOL

The QOL of the patients with cancer was measured using the Chinese-language translation of the validated European Organization for the Research and Treatment of Cancer QLQ-C30 (Quality of Life Questionnaire version 3.0) [33]. The QLQ-C30 includes a global health status or QOL scale [QL], 5 functional scales (physical [PF], role [RF], emotional [EF], cognitive [CF], and social [SF]), 3 symptom scales (fatigue, pain, and nausea and vomiting), and 6 single-item symptom scores. All scale scores range from 0 to 100, with a high score on the functional scales indicating a high level of functioning and a high score on the symptom scales indicating greater severity of individual symptoms.

#### Motion Sickness

The Virtual Reality Symptom Questionnaire (VRSQ), which was developed by Ames et al [34] in 2005, was used. After the VR intervention, the VRSQ was completed to evaluate the possible symptoms of motion sickness that may occur in a VR environment. The questionnaire assessed 8 general physical side effects (general discomfort, fatigue, boredom, drowsiness, headache, dizziness, concentration difficulties, and nausea) and 5 visual effects (eye fatigue, eye pain, blurred vision, and difficulty focusing). The score ranges from 0 to 6, indicating a degree of symptoms from none to very severe.

### Data Analysis

The analysis for this study was performed using IBM SPSS Statistics software (version 23). Appropriate descriptive statistics were used to summarize the characteristics and outcome variables of the participants. The *t* test, chi-square test, and the Wilcoxon signed-rank test were used to evaluate between-group differences. The intention-to-treat (ITT) principle was adopted for outcome analysis [35]. A generalized estimating equations (GEE) model is an extension of the quasi-likelihood method and is often used to analyze longitudinal data [36]. The characteristic of this data is the correlation between the multiple observation points for the same individual, which cannot be processed by the general linear model. The GEE model can solve the correlations between longitudinal data, as well as missing data. In this study, the GEE model was used to evaluate the effects of VR intervention on the changes of QOL and distress scores of patients with breast cancer at various time points (T0, T1, and T2). All statistical tests involved were 2-sided with the level of significance set at 0.05.

**Figure 2.**
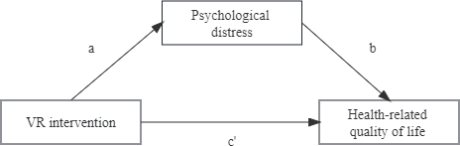
Hypothesized mediation model (a, b, and c′=unstandardized regression coefficients): indirect effect=a×b; direct effect=c′, total effect c=sum of indirect and direct effects=a×b+c′.

We used mediation analysis to investigate the relationship between the VR intervention at T0 and the health-related QOL at T2, with the psychological distress at T2 as a mediator (Figure 2). A mediator variable was defined as a third variable that changed the association between an independent and dependent variable [37]. It provided additional insight into information about the causal links between 2 strongly associated variables. The PROCESS macro with model 4 was used and, to ensure the stability of the path coefficient estimates, the analysis for the mediation model was supplemented with 5000 bootstrap replications. The significance of the indirect effect was examined by the bias-corrected 95% CI after bootstrapping. An indirect effect was considered statistically significant if the 95% CI did not include 0.

### Ethical Considerations

This study was performed in line with the principles of the Declaration of Helsinki. Approval was granted by the Ethics Committee of the Liaoning Cancer Hospital & Institute (reference 20200301-2). Each participant provided written informed consent to take part in the study. For the images with a participant' face and body (Figure 1), the author had obtained permission to publish in the study.

## Results

### Recruitment

**Figure 3.**
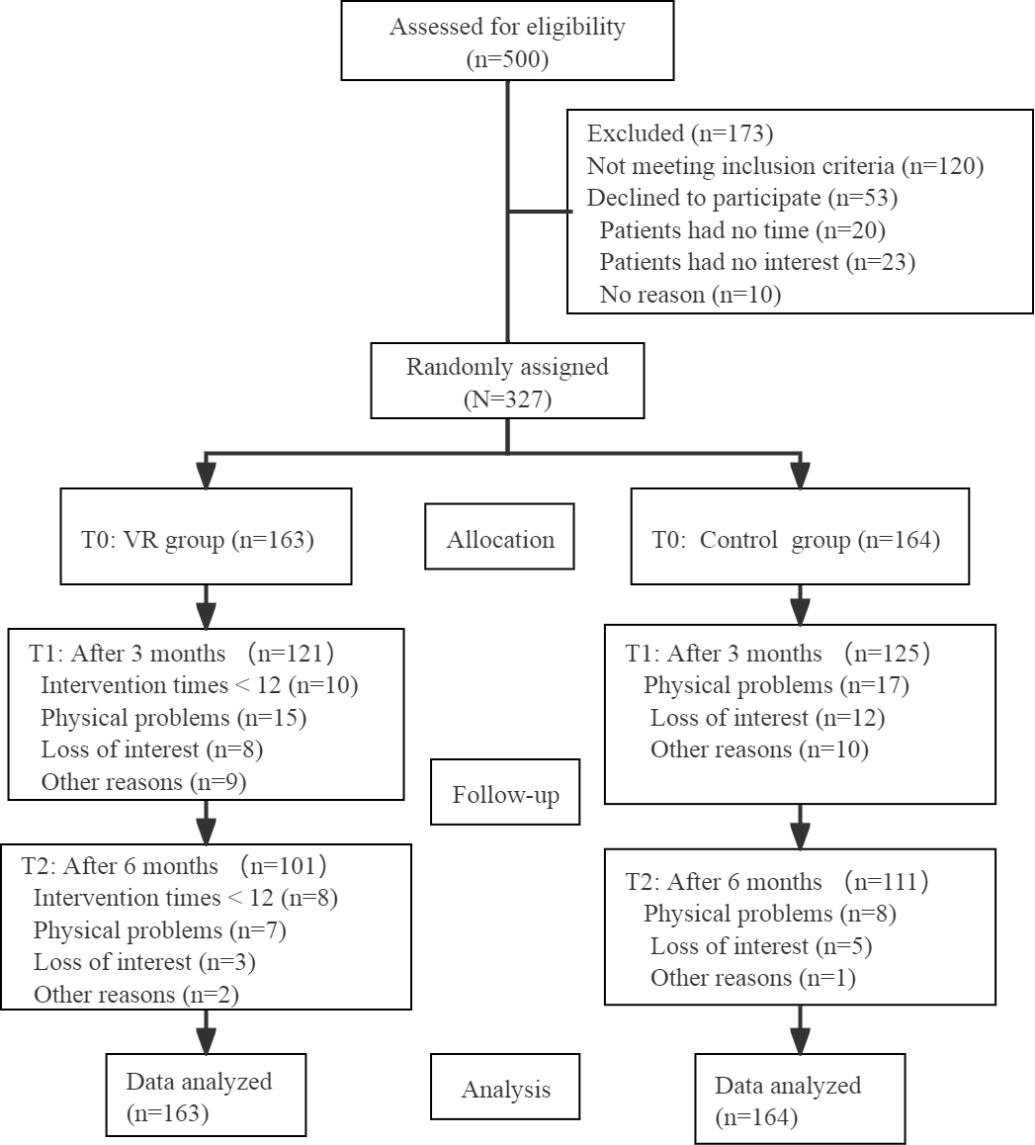
CONSORT diagram. CONSORT: Consolidated Standards of Reporting Trials; VR: virtual reality.

As shown in the CONSORT (Consolidated Standards of Reporting Trials) flow diagram (Figure 3; see checklist in Multimedia Appendix 1), 500 participants were screened for eligibility. Among these patients, 120 were excluded because they did not meet the criteria, and 53 declined to participate because of lack of time or interest, or for no specific reasons. Ultimately, 327 participants were recruited, although 97 participants failed to complete the reassessment at T1 or T2, and 18 participants did not have enough interventions. The T1 and T2 attrition rates were 25% and 14%, respectively, and the overall attrition rate was within our acceptable range. We compared the baseline characteristics between those who completed the study and those who dropped out from the study, and there were no significant differences (Multimedia Appendix 2).

### Demographics and Clinical Characteristics

The participants’ average age at diagnosis was 54.3 (SD 9.58) years, and of whom 216 (66.1%) were residents of a city, 198 (60.6%) had below middle school–level education, 267 (81.7%) were married, and 294 (89.9%) had no family history of cancer. More than half of the participants had an annual personal income of less than CN ¥20,000 (US $2838.61). In total, 263 (80.4%) participants had cancer at stage I or II. At the baseline, there were no differences between the 2 groups in either sociodemographic or clinical variables (Table 1).

**Table 1.** Demographic and clinical characteristics of participants by group at baseline.

Variables		Total (N=327)	VR^a^ (n=163)	Control (n=164)	Statistics	
					*t* test or chi-square (df)	*P* value
Age (years) at diagnosis, mean (SD)		54.3 (9.6)	53.6 (9.4)	55.2 (9.7)	0.852^b^ (325)	.09
**Residence, n (%)**					0.659^c^ (1)	.16
	City	216 (66.1)	114 (69.9)	102 (62.2)		
	Country	111 (33.9)	49 (30.1)	62 (37.5)		
**Education, n (%)**					1.001^c^ (2)	.99
	Below middle school	198 (60.6)	99 (60.7)	99 (60.4)		
	High school	79 (24.2)	39 (23.9)	40 (24.4)		
	University or above	50 (15.3)	25 (15.3)	25 (15.2)		
**Marital status, n (%)**					0.130^c^ (1)	.98
	Married	267 (81.7)	133 (81.6)	134 (81.7)		
	Single, widowed, or divorced	60 (18.3)	30 (18.4)	30 (18.3)		
**Work, n (%)**					0.349^c^ (2)	.84
	Yes	170 (52.0)	78 (47.9)	92 (56.1)		
	Retired	108 (33)	54 (33.1)	54 (32.9)		
	No	49 (15)	31 (19)	18 (11)		
**Menopause, n (%)**					1.963^c^ (1)	.18
	Yes	197 (60.2)	92 (56.4)	105 (64.0)		
	No	130 (39.8)	71 (43.6)	59 (36)		
**Family history of cancer, n (%)**					1.699^c^ (1)	.20
	Yes	33 (10.1)	20 (12.3)	13 (7.9)		
	No	294 (89.9)	143 (87.7)	151 (92.1)		
**Annual personal income (1 CN ￥=0.14 US $)**					2.422^c^ (2)	.30
	<20,000	181 (55.4)	85 (52.1)	96 (58.5)		
	20,000‐50,000	121 (37)	67 (41.1)	54 (32.9)		
	>50,000	25 (7.6)	11 (6.7)	14 (8.5)		
BMI (kg/m^2^), mean (SD)		25.6 (10.4)	24.4 (3.4)	24.9 (3.3)	1.366^b^ (325)	.17
**Cancer stage, n (%)**					3.869^c^ (3)	.51
	I	100 (30.6)	46 (28.2)	54 (32.9)		
	II	163 (49.8)	84 (51.5)	79 (48.2)		
	III	48 (14.7)	25 (15.3)	23 (14)		
	IV	16 (4.9)	8 (4.9)	8 (4.9)		

a VR: virtual reality.

b *t* test.

c Chi-square.

### Outcome Variables at Baseline

Overall, there was no significant difference between the 2 groups in each scale score at baseline (*P*<.05). In total, 245 (74.9%) patients scored ≥4 on the DT and had psychological distress. At the same time, participants in both groups reported low QOL at T0. Detailed scores are shown in Table 2.

**Table 2.** Mean scores of the outcome variables for intervention and control groups across study time points and the baseline comparisons.

Outcome variables andgroup		T0 (baseline),mean (SD)	T1 (3 months),mean (SD)	T2 (6 months),mean (SD)	Comparison of groups at T0	
					*t* test (*df*)	*P* value
**Participants (n)**						
	Control	164	125	111		
	VR^a^	163	121	101		
**DT** ^**b**^					1.306 (325)	.19
	Control	5.15 (2.35)	4.63 (1.91)	4.27 (2.21)		
	VR	4.83 (2.05)	3.27 (1.81)	3.01 (1.96)		
**QL** ^**c**^					−0.843 (325)	.40
	Control	46.87 (24.28)	55.12 (19.58)	54.12 (18.15)		
	VR	48.93 (19.49)	60.56 (17.95)	62.87 (22.10)		
**PF** ^**d**^					−0.392 (325)	.70
	Control	63.54 (23.08)	65.54 (22.57)	65.93 (20.71)		
	VR	64.54 (23.22)	72.63 (19.01)	80.20 (18.70)		
**RF** ^**e**^					−0.271 (325)	.79
	Control	62.91 (30.74)	66.33 (26.35)	68.31 (22.51)		
	VR	63.80 (29.14)	70.31 (27.35)	79.70 (24.56)		
**EF** ^**f**^					−1.108 (325)	.27
	Control	63.92 (19.14)	58.66 (24.03)	62.91 (26.07)		
	VR	66.36 (20.60)	74.76 (18.58)	79.62 (19.49)		
**CF** ^**g**^					−1.281 (325)	.20
	Control	71.85 (21.57)	65.18 (24.51)	67.40 (26.52)		
	VR	74.95 (22.17)	81.11 (17.91)	81.02 (20.42)		
**SF** ^**h**^					−1.776 (325)	.08
	Control	57.42 (27.27)	54.13 (26.18)	59.34 (21.55)		
	VR	62.88 (28.35)	63.97 (25.12)	72.94 (21.72)		

a VR: virtual reality.

b DT: Distress Thermometer.

c QL: global health status or quality of life scale.

d PF: physical functioning.

e RF: role functioning.

f EF: emotional functioning.

g CF: cognitive functioning.

h SF: social functioning.

### Effects of VR Interventions

#### Effects on Distress

**Figure 4.**
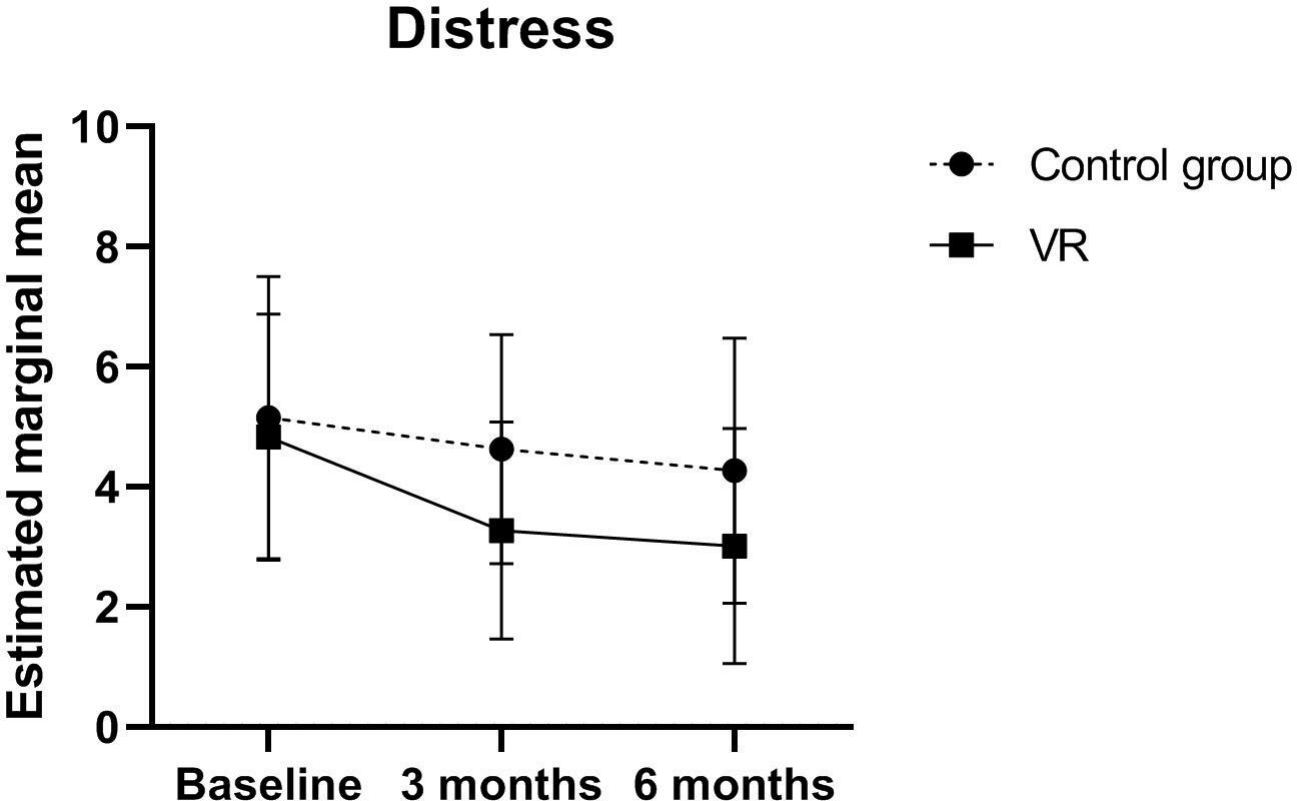
Mean scores of distress in the virtual reality and control groups at the 3 time points of data collection. VR: virtual reality.

As shown in Figure 4 and Table 3, compared with T0, DT scores in the VR group gradually decreased at T1 and T2 (T1: mean 3.27, SD 1.81; T2: mean 3.01, SD 1.96), and this change was significantly different from that in the control group, as indicated by the interaction term of group×T1 (β: −1.049, 95% CI −1.635 to −0.445; *P*=.001) and group×T2 (β: −.947, 95% CI −1.635 to −0.258; *P*=.007).

**Table 3.** Generalized estimating equation models for the comparison of outcomes across time between the control and intervention groups.

Outcome variables	Regression coefficients									
Group		T1^a^		T2^b^		Group×T1		Group×T2	
*β* (95% CI)	*P* value	*β* (95% CI)	*P* value	*β* (95% CI)	*P* value	*β* (95% CI)	*P* value	*β* (95% CI)	*P* value
DT^c^	−.318 (−.794, 0.158)	.19	−.513 (−0.972, −0.053)	.03	−.872 (−1.417, −0.326)	.002	−1.049 (−1.635, −0.445)	.001	−.947 (−1.635, −0.258)	.007
QL^d^	2.052 (−2.704, 6.808)	.40	8.242 (3.346, 13.137)	.001	7.247 (2.452, 12.042)	.78	3.388 (−3.023, 9.799)	.30	6.698 (−0.147, 13.543)	.06
PF^e^	1.003 (−3.999, 6.006)	.69	2.008 (−3.731, 7.747)	.49	2.397 (−3.343, 8.138)	.41	6.087 (−1.396, 13.570)	.11	13.261 (5.434, 21.087)	.001
RF^f^	.897 (−5.575, 7.370)	.79	3.43 (−3.220, 10.081)	.31	5.409 (−1.149, 11.966)	.11	3.084 (−5.873, 12.041)	.50	10.491 (1.419, 19.563)	.02
EF^g^	2.437 (−1.861, 6.735)	.27	−5.259 (−10.603, 0.084)	.05	−1.011 (−6.891, 4.870)	.74	13.661 (6.991, 20.332)	<.001	14.271 (7.084, 21.459)	<.001
CF^h^	3.099 (−1.629, 7.827)	.20	−6.668 (−12.323, −1.014)	.02	−4.45 (−10.566, 1.666)	.15	12.83 (5.928, 19.733)	<.001	10.525 (2.953, 18.096)	.006
SF^i^	5.465 (−0.547, 11.476)	.08	−3.239 (−9.473, 2.887)	.30	1.922 (−3.434, 7.278)	.48	4.378 (−3.725, 12.482)	.03	8.132 (0.479, 15.785)	.04

a T1: the third months of intervention.

b T2: the sixth months of follow-up.

c DT: Distress Thermometer.

d QL: global health status or quality of life scale.

e PF: physical functioning.

f RF: role functioning.

g EF: emotional functioning.

h CF: cognitive functioning.

i SF: social functioning.

#### Effects on Health-Related QOL

**Figure 5.**
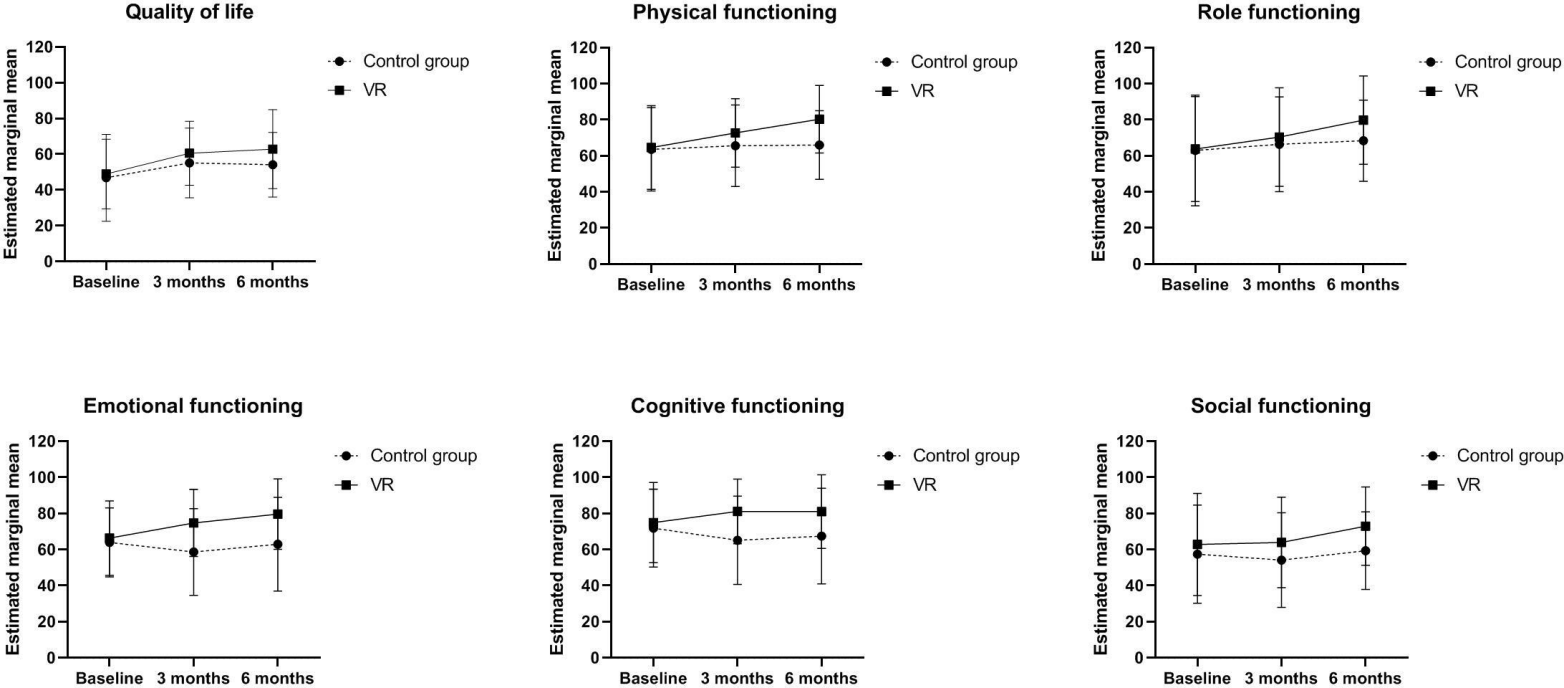
Mean scores on each dimension of quality of life in the virtual reality and control groups between the 3 time points of data collection. VR: virtual reality.

As shown in Figure 5, the VR group showed a great improvement in the global health status scale at T1 and T2 but, when compared with the control group, statistical significance was not reached (group×T1: *P*=.30 and group×T2: *P*=.06). In terms of functional subscales, significant differences between the changes in the 2 groups were observed in the PF and RF scales at T2 (*P*=.001 and .02), and in the EF, CF, and SF scales at both T1 and T2 (*P*<.05; Table 3). At the same time, we also analyzed the other symptom subscales. Nausea and vomiting, appetite loss, dyspnea, and other symptoms of participants in the VR group were also significantly improved at different time points with respect to T0 (*P*<.05), as shown in Multimedia Appendices 3 and 4.

#### Results of the Mediating Analysis

According to the GEE model, the VR group had significantly greater improvement in distress compared with the control group across the preintervention and postintervention time points. The differential changes between the 2 groups supported the further exploration of the mediating roles. Table 4 shows that psychological distress had a significant indirect effect on the global health status in the participants of the VR group (indirect effect=6.245; 95% CI 2.965, 10.282). Similarly, the effects of VR on 5 health-related functional subscales were also mediated by improving psychological distress (indirect effect=4.572‐6.672, *P*<.05).

**Table 4.** Mediation analysis results for the virtual reality effects on health-related quality of life via distress. The virtual reality intervention group at T0 was set as the independent variable (X), the global health status and 5 functioning subscales at follow-up T2 were set as dependent variables (Y), and the distress at T2 as a mediator (M). * *P*<.05.

Variables	a^a^	b^b^	c’^c^	ab^d^	95% CI
QL^e^	−1.265^*^	−4.938^*^	2.506	6.245^*^	2.965-10.282
PF^f^	−1.265^*^	−4.333^*^	8.784^*^	5.480^*^	2.645-8.725
RF^g^	−1.265^*^	−3.803^*^	6.578	4.810^*^	2.226-8.022
EF^h^	−1.265^*^	−5.275^*^	10.037^*^	6.672^*^	3.553-10.004
CF^i^	−1.265^*^	−3.615^*^	9.052^*^	4.572^*^	2.320-7.266
SF^j^	−1.265^*^	−3.724^*^	8.886^*^	4.711^*^	2.080-7.919

a a: the direct effect of X on M.

b b: the direct effect of M on Y when X is controlled.

c cʹ: the direct effect of X on Y when M is controlled.

d ab: the indirect effect of X on Y through M.

e QL: global health status or quality of life scale.

f PF: physical functioning.

g RF: role functioning.

h EF: emotional functioning.

i CF: cognitive functioning.

j SF: social functioning.

### Cybersickness Symptoms

Some cybersickness symptoms related to VR were analyzed through VRSQ. Data showed that in the first month of intervention, the frequency of patients with each symptom was less than 20% (32/163; Multimedia Appendix 5).

## Discussion

### Principal Findings

This study demonstrated that VR technology may help reduce distress and improve the health-related QOL of patients with breast cancer over time. By incorporating a mediation analysis, we showed that the QOL benefits of VR are manifested through its positive effects on psychological distress risk factors.

Psychological distress is a common side effect of cancer treatment. In 2018, a cross-sectional study completed by our team showed that 56.5% of cancer survivors had psychological distress and scored ≥4 on the DT [38]. However, the outbreak of COVID-19 aggravated the emotional distress of patients with cancer. In this study, the proportion rose to 74.9% (245/327) and it was necessary to take appropriate intervention measures to reduce the psychological pressure. VR allows patients to be completely engaged in an immersive environment, which may help distract them from noxious stimuli, thereby relieving psychological stress [19,28]. In our study, the distress level was significantly reduced in the VR group compared with the control group (undergoing usual care). This longitudinal effect did not disappear over time, suggesting that the effect of VR was not simply due to the novelty of the experience [22]. Yang et al [39] proposed a 4-layer theoretical framework of a potential VR intervention for mental health. Based on the hypothesis, VR delivers predictability in an unpredictable environment, and helps patients cope with their stress, evokes emotion, and diverts attention [40].

VR has shown promise in improving health status among patients with breast cancer [41]. Reynolds et al [30] reported that participants with metastatic breast cancer who were subjected to VR experiences showed clinically significant decreases in fatigue, stress, and pain during the trial and at follow-up. However, many studies focus on the efficacy of in-the-moment distraction from treatment, and the long-term effects of VR are still missing. Some of our preliminary findings are encouraging. The longitudinal effects of VR could be sustained at least for 6 months. At the same time, these positive effects were not only shown in the dimensions of PF and EF of QOL, but also in the dimensions of RF, CF, and SF, and even some chemotherapy side effects such as vomiting and insomnia were improved and relieved to varying degrees. This was also a comprehensive assessment of the effects of VR on QOL. In addition to the clear effect on pain management [42,43], this study also provided more possibilities for VR in improving patients’ QOL and chemotherapy tolerance. In the future, VR may be considered for incorporation into the clinical setting to provide a convenient, attractive, and easily applied intervention.

The psychological and physiological fields are the most commonly studied areas for VR [26,44]. In our study, we focused on relatively stable hospitalized patients undergoing chemotherapy rather than surgical patients. We agree with Espinoza et al [45] that VR technology shifted patients’ attention to the selected scene track, and reduced their attention to pain or anxiety. However, presumably this distraction effect did not occur in the days after the removal of the device, and so could not fully explain the maintenance of VR. Our other important finding was that psychological distress could play a mediating role in the VR-QOL interaction, which provides an explanation for the persistence of VR. Psychological factors play an important role in the development and regression of tumors [46]. Generally speaking, a good state of psychological health can stimulate a number of body functions and significantly boost the effectiveness of the chemotherapy [47]. Our results confirmed once again that improved mood can promote positive health outcomes and QOL. Several design characteristics of our VR system, such as guided relaxation and soothing music, support the mediation theory of positive mood. These observations further suggest that the intervention mechanism of VR is multifactorial, being mediated by attentional, cognitive, and emotional effects [48].

Owing to the specificity of the disease, the impact of the COVID-19, and the long study period, some participants dropped out of the study, and the overall attrition rate reached 35%, which was similar to previous studies [31] and within our estimated range. However, among the lost participants, only 18 patients were lost due to the substandard intervention frequency, accounting for less than 16%. At the same time, the VR equipment we used was comfortable to wear, and the designed scenes were natural. We also assessed the possible side effects of VR devices through the VRSQ scale, but all symptoms occurred at a frequency of less than 20%, which was considered negligible [49]. These findings are important and demonstrate that VR intervention can be a viable and acceptable treatment for patients with breast cancer [30].

### Limitations

Although this study had several strengths and extended the literature on the use of VR, it was not without limitations. First, loss of muscle strength leading to a decrease in limb functionality is also a common complication seen in breast cancer survivors. This VR system did not incorporate exercise rehabilitation training such as improving patients’ upper limb mobility, so the research on the effects of VR was incomplete. Second, although we designed 3 intervention scenarios, and required each patient to achieve multiple VR interventions, we did not further analyze the impact of the duration and frequency of interventions, and did not verify the effect of repeated VR exposure. Third, this study assessed the effects of VR through self-reported measures, but because the participants, investigators, and nurses were unlikely to be blind, this could have introduced bias into some of the results. In future, physiological indicators (such as stress-related molecules or brain waves) can be used to monitor changes and evaluate the effects of VR.

### Conclusions

This study found that VR has the potential to sustainably relieve the level of distress and improve the QOL in women with breast cancer. Importantly, the positive results were sustained for at least 6 months. Furthermore, reducing emotional distress was identified as one of the possible mechanisms by which VR affects QOL. As physicians, it is our duty to participate in the development of these innovations to meet the clinical need for effective nonpharmacologic adjuncts, especially for patients with cancer or during special times such as the pandemic. We suggest that future research should be carried out in multiple centers and medical institutions to verify the effectiveness with other cancer treatments and to explore more possibilities for VR interventions.

## Supplementary material

10.2196/53825 Multimedia Appendix 1 CONSORT-EHEALTH (Consolidated Standards of Reporting Trials of Electronic and Mobile Health Applications and Online Telehealth) checklist.

10.2196/53825 Multimedia Appendix 2 Demographic and clinical characteristics of participants based on completion status at baseline.

10.2196/53825 Multimedia Appendix 3 Mean scores on the symptom subscales for intervention and control groups across study time points and the baseline comparisons.

10.2196/53825 Multimedia Appendix 4 Generalized estimating equation models for the comparison of outcomes across time between the control and intervention groups.

10.2196/53825 Multimedia Appendix 5 Percentage frequency of the occurrence of each symptom in the Virtual Reality Symptom Questionnaire.

## Abbreviations

CF: cognitive functioning
CONSORT: Consolidated Standards of Reporting Trials
DT: Distress Thermometer
EF: emotional functioning
GEE: generalized estimating equation
ITT: intention-to-treat
PF: physical functioning
QL: global health status or quality of life scale
QLQ-C30: Quality of Life Questionnaire version 3.0
QOL: quality of life
RF: role functioning
SF: social functioning
VR: virtual reality
VRSQ: Virtual Reality Symptom Questionnaire
