# Correction: Effects of Virtual Reality Therapy for Patients With Breast Cancer During Chemotherapy: Randomized Controlled Trial

**DOI:** 10.2196/67718

**Published:** 2024-11-06

**Authors:** Mengdan Li, Zhifu Yu, Hui Li, Li Cao, Huihui Yu, Ning Deng, Yunyong Liu

**Affiliations:** 1 Cancer Hospital of China Medical University, Liaoning Cancer Hospital & Institute, ShenYang, China; 2 National Cancer Center, National Clinical Research Center for Cancer, Cancer Hospital & Shenzhen Hospital, Chinese Academy of Medical Sciences and Peking Union Medical College, Shenzhen, China

In “Effects of Virtual Reality Therapy for Patients With Breast Cancer During Chemotherapy: Randomized Controlled Trial” (JMIR Serious Games 2024;12: e53825) the authors noted two errors.

Affiliation 2 was previously listed as:

National Cancer Center, National Clinical Research Center for Cancer, Cancer Hospital & Shenzhen Hospital

This has been changed to the following:

National Cancer Center, National Clinical Research Center for Cancer, Cancer Hospital & Shenzhen Hospital, Chinese Academy of Medical Sciences and Peking Union Medical College

As this affiliation was also included in the corresponding author’s address, the address has been modified in that location as well.

Additionally, the authorship’s degrees were previously listed as:

Mengdan Li, MD; Zhifu Yu, MD; Hui Li, MD; Li Cao, BM; Huihui Yu, MD; Ning Deng, PhD; Yunyong Liu, MD

These have been replaced as follows:

Mengdan Li, MPH; Zhifu Yu, MPH; Hui Li, MSN; Li Cao, BSN; Huihui Yu, MPH; Ning Deng, MD; Yunyong Liu, MM

The correction will appear in the online version of the paper on the JMIR Publications website on November 6, 2024, together with the publication of this correction notice. Because this was made after submission to PubMed, PubMed Central, and other full-text repositories, the corrected article has also been resubmitted to those repositories.

