# Supplementary material for: Effects of Virtual Reality Therapy for Patients With Breast Cancer During Chemotherapy: Randomized Controlled Trial
Source: JMIR Serious Games. 2024 Oct 17;12:e53825. doi: 10.2196/53825 (PMC11500621; doi:10.2196/53825)
Supplement: Multimedia Appendix 2 [file games-v12-e53825-s002.docx]

| **Variables** | **Incomplete (n=115)** | | **Complete (n=212)** | | **Statistics** | |
| --- | --- | --- | --- | --- | --- | --- |
|  | **N** | **%** | **N** | **%** | **t/χ^2^** | ***P*** |
| Age at diagnosis (years),  [mean (SD)] | 54.0 | 9.8 | 54.5 | 9.4 | 0.512 | .61 |
| Residence |  |  |  |  | 0.940 | .39 |
| City | 72 | 62.6 | 144 | 67.9 |  |  |
| Country | 43 | 37.4 | 68 | 32.1 |  |  |
| Education |  |  |  |  | 0.668 | .72 |
| Below middle school | 67 | 58.3 | 131 | 61.8 |  |  |
| High school | 28 | 24.3 | 51 | 24.1 |  |  |
| University or above | 20 | 17.4 | 30 | 14.2 |  |  |
| Marital Status |  |  |  |  | 0.109 | .77 |
| Married | 95 | 82.6 | 172 | 81.1 |  |  |
| Single/widowed/divorced | 20 | 17.4 | 40 | 18.9 |  |  |
| Work |  |  |  |  | 2.961 | .23 |
| Yes | 65 | 56.5 | 105 | 49.5 |  |  |
| Retire | 31 | 27.0 | 77 | 36.3 |  |  |
| No | 19 | 16.5 | 30 | 14.2 |  |  |
| Menopause |  |  |  |  | 3.841 | .06 |
| Yes | 61 | 53.0 | 136 | 64.2 |  |  |
| No | 54 | 47.0 | 76 | 35.8 |  |  |
| Family history of cancer |  |  |  |  | 0.521 | .53 |
| Yes | 12 | 10.4 | 13 | 9.9 |  |  |
| No | 103 | 89.6 | 151 | 90.1 |  |  |
| Annual person income (CNY) |  |  |  |  | 3.616 | .16 |
| <20,000 | 69 | 60.0 | 112 | 52.8 |  |  |
| 20,000-50,000 | 35 | 30.4 | 86 | 40.6 |  |  |
| >50,000 | 11 | 9.6 | 14 | 6.6 |  |  |
| BMI (kg/m^2^), [ mean (SD)] | 24.9 | 3.4 | 24.5 | 3.4 | -0.908 | .37 |
| Cancer stage |  |  |  |  | 0.294 | .96 |
| Ⅰ | 34 | 29.6 | 66 | 31.1 |  |  |
| Ⅱ | 58 | 50.4 | 105 | 49.5 |  |  |
| Ⅲ | 18 | 15.7 | 30 | 14.2 |  |  |
| Ⅳ | 5 | 4.3 | 11 | 5.2 |  |  |

Abbreviations: VR, Virtual Reality; SD, standard deviation; CNY, Chinese Yuan; BMI, Body Mass Index.
