# Supplementary material for: Effects of Virtual Reality Therapy for Patients With Breast Cancer During Chemotherapy: Randomized Controlled Trial
Source: JMIR Serious Games. 2024 Oct 17;12:e53825. doi: 10.2196/53825 (PMC11500621; doi:10.2196/53825)
Supplement: Multimedia Appendix 3 [file games-v12-e53825-s003.docx]

| **Outcome variables** | **Group** | **T0 (baseline)**  **Mean (SD)** | **T1 (3 months)**  **Mean (SD)** | **T2 (6 months)**  **Mean (SD)** | **Comparison of groups at T0** | | |
| --- | --- | --- | --- | --- | --- | --- | --- |
|  |  |  |  |  | **t** | | ***P*** |
| FA | Control | 43.97 (23.33) | 40.37(24.98) | 38.58 (23.09) | 2.564 | .01 | |
|  | VR | 37.21 (24.21) | 27.40 (24.56) | 24.64 (21.29) |  |  | |
| NV | Control | 16.67 (21.38) | 32.67 (26.66) | 34.80 (26.60) | 0.675 | .50 | |
|  | VR | 15.03 (22.47) | 15.24 (18.65) | 16.67 (21.08) |  |  | |
| PA | Control | 33.64 (26.14) | 26.40 (25.96) | 26.19 (25.23) | 1.964 | .05 | |
|  | VR | 28.22 (23.67) | 17.78 (21.34) | 13.70 (19.06) |  |  | |
| DY | Control | 20.12 (24.10) | 30.03 (31.80) | 33.33 (33.33) | 1.563 | .12 | |
|  | VR | 16.16 (21.71) | 12.06 (20.21) | 12.54 (21.53) |  |  | |
| SL | Control | 36.59 (30.94) | 32.67 (29.05) | 35.16 (31.18) | 1.660 | .10 | |
|  | VR | 31.08 (28.96) | 24.44 (27.06) | 22.11 (26.78) |  |  | |
| AP | Control | 27.03 (26.26) | 34.98 (25.56) | 34.43 (28.31) | 0.427 | .67 | |
|  | VR | 25.77 (27.29) | 25.08 (24.80) | 27.72 (23.60) |  |  | |
| CO | Control | 19.51 (25.56) | 27.72 (29.47) | 28.94 (29.48) | 0.780 | .44 | |
|  | VR | 17.38 (23.81) | 11.74 (19.05) | 13.20 (19.48) |  |  | |
| DI | Control | 11.38 (20.37) | 26.40 (28.01) | 24.18 (26.32) | 1.204 | .23 | |
|  | VR | 8.79 (18.45) | 11.11 (22.96) | 8.91 (16.93) |  |  | |
| FI | Control | 45.73 (33.67) | 43.89 (29.03) | 34.80 (25.78) | 0.599 | .55 | |
|  | VR | 43.56 (31.93) | 39.05 (30.12) | 30.03 (27.29) |  |  | |

Abbreviations: FA, Fatigue; PA, Pain; DY, Dyspnoea; SL, Sleep disturbance; AP, Appetite loss; CO, Constipation; DI, Diarrhoea; FI, Financial impact; VR, Virtual Reality; SD, standard deviation.
