# Supplementary material for: Effects of Virtual Reality Therapy for Patients With Breast Cancer During Chemotherapy: Randomized Controlled Trial
Source: JMIR Serious Games. 2024 Oct 17;12:e53825. doi: 10.2196/53825 (PMC11500621; doi:10.2196/53825)
Supplement: Multimedia Appendix 4 [file games-v12-e53825-s004.docx]

| **Outcomes** | **Regression Coefficients** | | | | | | | | | | | | | | |
| --- | --- | --- | --- | --- | --- | --- | --- | --- | --- | --- | --- | --- | --- | --- | --- |
|  | **Group** | |  | **T1** | |  | **T2** | |  | **Group×T1** | |  | **Group×T2** | | |
|  | ***β* (95% CI)** | ***P*** |  | ***β* (95% CI)** | ***P*** |  | ***β* (95% CI)** | ***P*** |  | ***β* (95% CI)** | ***P*** |  | ***β* (95% CI)** | ***P*** |  |
| FA | -6.751 (-11.306, -1.606) | .01 |  | -3.596 (-9.410, 2.218) | .23 |  | -5.387 (-11.306, 0.533) | .07 |  | -6.215 (-13.601, 1.170) | .10 |  | -7.190 (-14.845, 0.465) | .07 |  |
| NV | -1.636 (-6.375, 3.103) | .50 |  | 16.007 (9.787, 22.227) | <0.001 |  | 18.132 (11.677, 24.586) | <0.001 |  | -15.800 (-23.172, -8.426) | <0.001 |  | -16.500 (-24.704, -8.288) | <0.001 |  |
| PA | -5.418 (-10.840, 0.004) | .05 |  | -7.134 (-13.239, -1.029) | .02 |  | -7.346 (-14.097, -0.596) | .03 |  | -3.207 (-10.958, 4.545) | .42 |  | -7.076 (-15.454, 1.302) | .10 |  |
| DY | -3.763 (-8.755, 1.228) | .14 |  | 10.114 (3.038, 17.191) | .005 |  | 13.415 (5.578, 21.251) | .001 |  | -14.208 (-22.646, -5.767) | .001 |  | -17.029 (-26.391, -7.667) | <0.001 |  |
| SL | -5.502 (-11.977, 0.974) | .10 |  | -3.912 (-11.012, 3.188) | .28 |  | -1.421 (-9.242, 6.401) | .72 |  | -2.727 (-11.754, 6.300) | .54 |  | -7.511 (-17.538, 2.435) | .14 |  |
| AP | -1.266 (-7.053, 4.522) | .67 |  | 7.951 (1.662, 14.240) | .01 |  | 7.400 (0.677, 14.123) | .03 |  | -8.638 (-16.976, -0.300) | .04 |  | -5.444 (-14.437, 3.550) | .24 |  |
| CO | -2.130 (-7.467, 3.207) | .43 |  | 8.211 (1.176, 15.245) | .02 |  | 9.426 (2.343, 16.508) | .009 |  | -13.847 (-22.131, -5.563) | .001 |  | -13.607 (-21.936, -5.278) | .001 |  |
| DI | -2.589 (-6.788, 1.611) | .23 |  | 15.021 (8.876, 21.166) | <0.001 |  | 12.794 (6.588, 18.999) | <0.001 |  | -12.703 (-20.450, -4.956) | .001 |  | -12.676 (-20.141, -5.211) | .001 |  |
| FI | -2.582 (-9.754, 4.589) | .48 |  | -1.837 (-8.737, 5.063) | .60 |  | -10.933 (-17.883, -3.983) | .002 |  | -2.264 (-11.420, 6.891) | .63 |  | -2.183 (-11.507, 7.140) | .65 |  |

Abbreviations: T1, the third months of intervention; T2, the sixth months of follow-up; CI, confidence interval; FA, Fatigue; PA, Pain; DY, Dyspnoea; SL, Sleep disturbance; AP, Appetite loss; CO, Constipation; DI, Diarrhoea; FI, Financial impact.
