# Supplementary material for: Effects of Virtual Reality Therapy for Patients With Breast Cancer During Chemotherapy: Randomized Controlled Trial
Source: JMIR Serious Games. 2024 Oct 17;12:e53825. doi: 10.2196/53825 (PMC11500621; doi:10.2196/53825)
Supplement: Multimedia Appendix 5 [file games-v12-e53825-s005.docx]

|  | **None** | **Slight** | | **Moderate** | | **Severe** | |
| --- | --- | --- | --- | --- | --- | --- | --- |
| **Symptom** | **0** | **1** | **2** | **3** | **4** | **5** | **6** |
| General discomfort | 90.0 | 6.7 | 3.3 | 0 | 0 | 0 | 0 |
| Fatigue | 96.7 | 3.3 | 0 | 0 | 0 | 0 | 0 |
| Boredom | 96.7 | 0 | 3.3 | 0 | 0 | 0 | 0 |
| Drowsiness | 73.3 | 10.0 | 6.7 | 6.7 | 3.3 | 0 | 0 |
| Headache | 96.7 | 3.3 | 0 | 0 | 0 | 0 |  |
| Dizziness | 93.4 | 3.3 | 3.3 | 0 | 0 | 0 | 0 |
| Difficulty Concentrating | 96.7 | 3.3 | 0 | 0 | 0 | 0 | 0 |
| Nausea | 100.0 | 0 | 0 | 0 | 0 | 0 | 0 |
| Tired eyes | 73.3 | 16.7 | 6.7 | 3.3 | 0 | 0 | 0 |
| Aching eyes | 96.7 | 3.3 | 0 | 0 | 0 | 0 | 0 |
| Eyestrain | 73.3 | 16.7 | 6.7 | 3.3 | 0 | 0 | 0 |
| Blurred | 73.3 | 13.3 | 6.7 | 6.7 | 0 | 0 | 0 |
| Difficulties Focusing | 96.7 | 3.3 | 0 | 0 | 0 | 0 | 0 |
